# Supplementary material for: HDAC6 Regulates Radiosensitivity of Non-Small Cell Lung Cancer by Promoting Degradation of Chk1
Source: Cells. 2020 Oct 4;9(10):2237. doi: 10.3390/cells9102237 (PMC7600810; doi:10.3390/cells9102237)
Supplement: Supplementary file 1 [file cells-09-02237-s001.pdf]

# Supplemental Materials

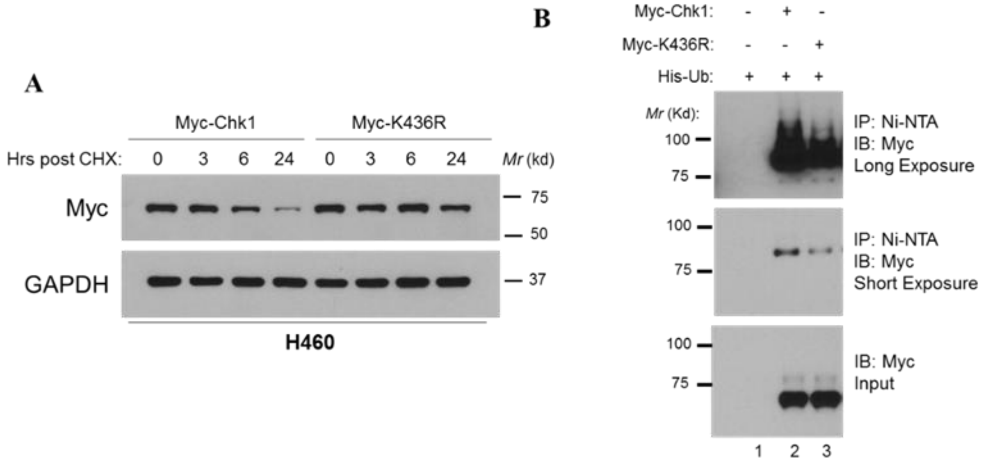


**Figure S1. Lysine 436 contributes to Chk1 stability**. (**A**) The indicated Myc-tagged Chk1 constructs were transfected into H460 cells. 48 h post-transfection, cells were treated with 150 μg/mL cycloheximide (CHX) and harvested at the indicated times. Lysates were analyzed via Western blot. (**B**) The indicated constructs were transfected into 293T cells. 48 h post-transfection, cells were harvested and lysates were probed with Ni-NTA beads for His-tagged protein under denatured conditions. The immunoprecipitated proteins were run on an SDS-PAGE gel, and probed for anti-Myc.

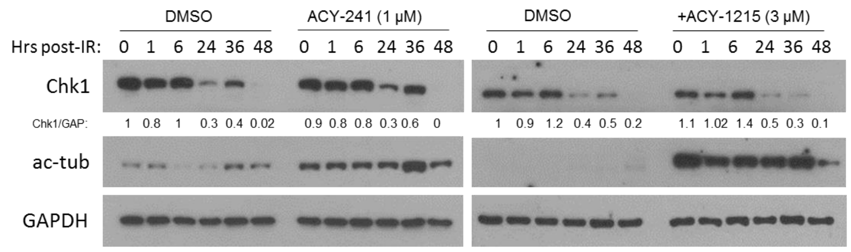


**Figure S2. Inhibition of HDAC6's deacetylase activity does not increase Chk1 protein stability post-IR.** A549 cells were treated for 48 h with either DMSO, ACY-241, or ACY-1215. Cells were then irradiated with 10 Gy radiation, harvested at the indicated time points. The lysates were analyzed by Western blot with indicated antibodies. The Chk1 bands were quantified by ImageJ.
